# The constructive power of informality? Relationships, emotion, and empathy in the administration of social assistance for childhood disability in South Africa

**DOI:** 10.1016/j.socscimed.2025.118211

**Published:** 2025-05-23

**Authors:** Zara Trafford

**Affiliations:** a Psychology Department, Stellenbosch University, South Africa; b Institute for Life Course Health Research, Department of Global Health, Stellenbosch University, South Africa

## Abstract

Social assistance cash transfers, known locally as grants, are the only regular financial support available to low-income families in South Africa. There are two broad categories: poverty alleviation and disability-related grants. All disability-related grants are linked with the public sector health system, because only medical doctors are permitted to conduct the required assessments. This article reflects on a three-year qualitative study of the perspectives of stakeholders around the central focus of the Care Dependency Grant for primary caregivers of disabled children. In this article, various framings of disability-related grants are unpacked, with a specific focus on the gatekeeping practices of frontline social security officials in South Africa. I situate their behaviours within local and global conversations about systems for disability benefits assessment and distribution, which increasingly aim to suppress bureaucratic “emotionality” for fear that this produces subjective – and thus, unfair – decision-making. The latter imperative is intensified in South Africa because of justified concerns about corruption. In an under-resourced bureaucracy catering to a large population, however, high levels of informality are likely to persist. Building on Rupert Hodder’s concept of positive informality, I share examples of “constructive” informality from the abovementioned study and argue for the importance of closely examining and learning from these instances. I conclude by suggesting that instead of trying to suppress their humanity, public servants’ engagement with the populace could be enhanced by investing in their professional development toward increased empathy and relationship-building. This might be an untapped complementary approach to improving social justice in redistributive efforts.

## 1 Introduction

Disability-related social assistance cash transfers and programming are key facilitators of access to critical health and other support services for disabled people, especially in low- and middle-income countries (LMICs) (Côte, 2021; Hanass-Hancock and McKenzie, 2017; Ullmann et al., 2021; Wapling et al., 2021). This article reflects on selected findings from a multi-stakeholder qualitative study to investigate the Care Dependency Grant (CDG), one of South Africa’s unconditional social assistance cash transfers. Cash transfers are known locally as “grants” or “social grants”. The CDG, specifically, is available to the primary caregiver of a child who, “due to his or her physical or mental [sic] disability, requires and receives permanent care or support services” (DSD, 2022, p. 16; South African Parliament, 2004).

The CDG is in place to contribute to the costs involved in caring for a disabled child for families who fall under a specific income threshold (Trafford and Swartz, 2021). Disability can result in higher costs of living associated with support for an impairment (“direct costs”), as well as a range of potential “indirect costs”, such as the need to stay home from income-generating work to provide care (Hanass-Hancock et al., 2017; Mishra and Mishra, 2014; Mitra et al., 2017). For families who successfully navigate the onerous application procedure, the CDG can contribute to the cost of access to important health, educational, and other disability-related supports (including transport to health facilities, specialised nutritional support, assistive devices, and private occupational and similar therapies), although it falls far short of sufficiently covering these costs. The CDG is greatly appreciated by those who manage to gain access, as it is also the only source of regular, state-funded financial support for disabled children from lower income families in South Africa (Letsie, 2016; Trafford, 2023a). The CDG is insufficient for managing the considerable costs often associated with childhood disability but in the context of weak complementary service provision, it is a critical government intervention to support health, wellbeing, participation and access for this population.

Despite their importance for moderating the significant direct and indirect costs of disability, however, disability-related social assistance programmes are often a site of tension in popular media and governmental discourses both locally and internationally (Porter et al., 2021). In LMICs such as South Africa, these discourses are further complicated by high levels of poverty, a relatively small percentage of the population being able to contribute to the fiscus via income tax, concerns about corruption within government, and widespread misunderstandings about the varied manifestations of disability (N. Gray, 2021; Hansen and Sait, 2011; G. Kelly, 2013; Naidoo, 2013; Pillay, 2017; Spyromitros and Panagiotidis, 2022). South Africa’s public sector service delivery systems are also generally overloaded, under-resourced, and plagued by maladministration, inefficiency, and corruption (Francis and Webster, 2019; Salahuddin et al., 2020).

This article begins with a detailed discussion of the preoccupation with fraud at the South African Social Security Agency (SASSA), a government “implementation agency” which administers all social assistance grants in the country. I contrast this focus on fraud with an apparent lack of commensurate attention to disability rights and inclusion. Next, I offer a brief introduction to Rupert Hodder’s concept of “positive informality” (Hodder, 2010, 2016, 2017a). Drawing specifically on his definition of informality as “social relationships, emotion [s], and the unwritten norms and conventions that regulate them” (Hodder, 2017, p. 18), I consider the extent to which these features of informality may enhance the functioning of public services toward their ultimate policy goals, particularly in the low-resource environment within which our government bureaucracies operate. This is in contrast with more common approaches to governance, which suggest that increased formality leads inevitably to improved fairness and efficacy. Finally, I provide a series of short vignettes from the wider study that produced these insights. These vignettes describe some examples of the informal behaviours of and relationships formed by government workers involved in disability-related grant administration, as well as other public servants who work to support children with disabilities and their families.

In this article, I suggest that rather than attempting to suppress the “emotionality” of under-resourced bureaucratic systems for fear of allowing too much space for corruption, we may learn something new and valuable by acknowledging that the relationships that are constructed informally have the power to yield constructive outcomes, as well as their more publicised destructive effects. The article concludes with a consideration of how frontline public servants’ engagement with disability-related grant clients may be enhanced by investing in their training and professional development, with potential for improvements in access and human rights for disabled children and their families. While this article is specific to the South African context, the insights are relevant for similar socioeconomic settings, as well as in other settings that are markedly different but may have something to learn from South Africa (Holt, 2024).

## 2 Materials and methods

Ethical approval for the wider Doctoral study reflected upon in this article was obtained from the Stellenbosch University Research Ethics Committee: Social Behavioural and Education Research (REC: SBE13097) with additional approvals from SASSA, the national Department of Social Development (DSD), and the Western Cape provincial Department of Health (WCDoH). Data collection involved indepth individual interviews with stakeholders including officials working at SASSA (at all levels from frontline clerks to office and regional managers and up to national leadership), doctors who conduct disability assessments for the CDG and in this province are employed by WCDoH, caregivers currently or formerly in receipt of the CDG, and officials from the national DSD, the government department that designed the Social Assistance Act, which governs grants distribution and eligibility in the country.

Most data were collected from participants in the Western Cape, one of South Africa’s nine provinces, and these data were complemented with interviews with participants from three other provinces and officials from national government. Between November 2020 and April 2022, I combined purposive and snowball sampling to recruit participants, usually via a brief introductory email or telephone conversation. Participants who expressed interest were sent an ethically-approved informed consent form over email, and consent was recapped at the beginning and end of each interview. Barring one focus group discussion at study end and reported on elsewhere (Trafford, 2023a), data collection was remote via a secure videoconferencing platform. For direct quotes below, I have denoted SASSA officials as “SO” and other participants as “KI” (key informant), with a numeral reflecting when each participant was interviewed (i.e., “SO4” referring to the SASSA participant who was interviewed fourth). I used pseudonyms for participants whose narratives were shared in more detail in the vignettes toward the end of this article.

In addition to these interviews, I conducted three years of complementary desk-based research. The latter included an extensive literature review, a detailed historical analysis of the origins and evolution of the CDG from the 1970s to date (using legislation and associated regulations, as these have not been documented in full), and reviews of SASSA’s internal guidelines and disability grant assessment forms. Previous publications from the same study have shared additional methodological details and participant demographic information, as well as findings relating to the policies associated with and bureaucratic administration of the CDG from the perspective of officials who administer these grants (Trafford and Swartz, 2021), doctors who conduct eligibility assessments (Trafford and Swartz, 2022), and caregivers who are currently or were previously in receipt of the CDG (Trafford, 2023a). Another publication from this series also documents conventions that protect the rights of the child and the rights of disabled people and considers whether the implementation of the CDG is currently aligned with these international guidelines (Trafford, 2023b).

In this article, I have not reproduced the methodological details of this study in depth, to avoid publishing the same work multiple times; fuller details of these processes can be found in the above articles. For this paper, I reflected on summary insights gained from the wider Doctoral study, for which inductive and deductive coding were combined to conduct an iterative and reflexive thematic analysis, as documented by Braun and Clarke (2019). I conducted all data collection and verbatim transcription myself, so data familiarisation occurred throughout the research process. Depth of understanding grew in conversation with my supervisor. In the earlier phases of the study, two descriptive papers reported on emerging insights from different participant groups and used deductive coding with a codebook based on the original research question, which aimed to investigate stakeholder understandings of the purpose of the CDG. This process was supported by Atlas.ti. In the later phases of analysis, two publications synthesised themes across participant groups and the existing literature, again using reflective thematic analysis as the basis of my analytical approach. In all instances, participants who were interested in reviewing their transcripts and/or the papers emerging from their narratives were given the opportunity to edit or clarify these transcripts and any direct quotes used in publications. Finally, during the completion of additional doctoral thesis chapters, deep engagement with and reflections on the data and the wider context in which it was gathered were documented, drawing especially on theory from disabled children’s childhood studies and the psychoanalysis of disability stigma. I have a decade of experience conducting qualitative and ethnographic research at the intersection of poverty and access to health services, working closely with lay health workers and low-income communities. These perspectives have influenced the ways in which I received and understood the data collected toward this study and have contributed to the ideas presented in this article.

In the conceptualisation and execution of the parent study, I was also influenced by Michael Lipsky’s theorisation of “street-level bureaucracy” (Lipsky, 1971, 2010), and Gabby Kelly’s important South African work investigating the role of doctors who assess applicant eligibility for the adult Disability Grant (DG) (G. Kelly, 2016b, 2017, 2019). In this article, I hope to add to a growing body of research that takes seriously the subjective experiences of frontline government officials, whose daily tasks and behaviours can shape the implementation and local instantiation of high-level policy in ways that are often destructive, but which may also be progressive.

## 3 SASSA guidelines for the administration of disability-related social assistance

South Africa was an early adopter of “rights-based” policies for health access and social assistance for disabled people (A. Gray and Vawda, 2020; Philpott and Muthukrishna, 2019; Schnitzler, 2020). In 2006, SASSA was established as an “implementation agency” for the DSD and since then, the Agency has been responsible for grant applications, their approval or rejection, and the distribution processes associated with all of the country’s social assistance cash transfers (Trafford and Swartz, 2021). These transfers include a range of poverty-alleviation grants: the child support grant for caregivers of children living in very low-income households, the old age grant for people older than 60 who do not have other sources of income, the war veteran’s grant, the foster care grant, and the social relief of distress grant, which recently exploded in beneficiary numbers due to the COVID19 pandemic. There are also grants associated with disability, which include the above-mentioned adult DG, the Grant-in-Aid (available to recipients of the DG and the old age grant who need additional care at home), and the CDG.

The historical and current application processes associated with what SASSA terms “disability-related grants” are described fully in related publications (Trafford and Swartz, 2021, 2022; Trafford, 2023a). The reality of this process varies widely across the country and in different municipalities but in short, an applicant must first visit a SASSA office to submit their application and identity documents. In the case of an adult DG, the applicant is an adult with a disability, and in the case of the CDG, the applicant is the primary caregiver of a child with a disability. A SASSA official should then make an appointment for the applicant or their child to undergo an eligibility assessment with a directly-contracted doctor or a doctor working in the public sector, in one province. Next, the eligibility assessment form should be transported by SASSA to the closest local office. The applicant should then be notified and return to the local SASSA office for their application outcome. At this point, if they have not yet submitted their financial documents, these should also be submitted for review. The final decision is made based on both the medical assessment and the applicant’s financial information. This information is processed by a supervisor, who can approve or deny the application based on threshold guidelines and the doctor’s decision.

The same administrators who process applications for the CDG also work with adult DG applicants (see below for further detail on this issue). Similarly, in eight of the country’s nine provinces, doctors are directly contracted by SASSA to conduct adult DG and CDG assessments. In the Western Cape province, there are a small number of these directly-contracted doctors, but most eligibility assessments are conducted in the public sector health system through a service-level agreement with the provincial Department of Health (Kelly, 2017; Trafford and Swartz, 2022). While specific arrangements vary according to the location, human resources for the administration and assessment of all disability-related grants are shared amongst the different disability-related grant types.

*Circa* 2002–2008, adult DG beneficiary numbers in South Africa grew rapidly. There was considerable concern about fraudulent access by the population but investigations at the time suggested that while fraud may have contributed in part to this uptick in numbers, the rising DG numbers were mostly due to broader circumstances. These circumstantial factors included soaring unemployment, a far larger population gaining entitlement to welfare benefits following the deracialisation of state-funded support post-apartheid, and many people being unable to work due to late-stage HIV progression before the 2004 launch of the public antiretroviral treatment programme (Delany et al., 2005). CDG numbers also rose in this period but at a lower rate, increasing from about 90,000 beneficiaries in 2006 to 110,000 in 2011, 120,000 in 2014, and 161,000 beneficiaries by October 2024 (SASSA, 2024). Inparallel, there have been ongoing and widely-publicised abuses of state funds throughout government, a pattern which intensified during the presidential administration in power from 2009 to 2018 (N. Gray, 2021; Salahuddin et al., 2020). Media and official reports of fraud internally at SASSA have also proliferated, with issues ranging from unethical agreements with private payment partners to the hoarding of multiple beneficiary cards in order to access funds illegally (Abrahams, 2024; Cedras and Gosai, 2024a; Damons, 2024a; A. du Toit, 2017; M. Du Toit and Lues, 2022; Gronbach, 2020; South African Parliament, 2022; Vally, 2016).

These issues, understandably, appear to have led to a fixation on fraud prevention in SASSA’s core guidelines and mission. The administration of all of the country’s disability-related grants is guided by the “Social Grants Disability Management Model” (SGDMM), which was first rolled out in 2009 and later updated, most recently in 2020 (SASSA Disability Management Unit, 2020). The SGDMM describes standard operating procedures for administering disability-related grant management and defines roles, structures, and functions within SASSA. This document also provides summaries of the legislative frameworks that govern SASSA’s work in disability-related grants management and details the challenges and risks involved in managing this portfolio. According to the SGDMM, when illegitimate applicants gain access to grants, this is “an administrative efficiency issue” (SASSA Disability Management Unit, 2020, p. 26). This issue is framed within the guideline as being responsible for “millions [of legitimate applicants] being denied access” (SASSA Disability Management Unit, 2020, p. 27). The best way to combat such fraud is apparently to “ensure [the] standardization and uniformity of all disability related processes” (SASSA Disability Management Unit, 2020, p. 2). In addition, supervisors are directed to observe their staff closely to avoid any fraudulent or inappropriate behaviour among SASSA clerical and administrative staff. Both “internal” and “external” fraud are thus identified as key threats to SASSA’s work in disability-related grants management.

In notable contrast to this prominent focus on limiting fraud, the SGDMM does not emphasise the critical importance of these grants for their key intention: sustaining the health and wellbeing of disabled children (and adults). The SGDMM *does* briefly discuss the prevalence and nature of disability in South Africa, but only as a preamble to the subsequent claim that there are currently too many “inclusion errors” among disability-related grant beneficiaries. The SGDMM also notes that because of the high demand for disability assessments and limited resources, SASSA has had to “implement unpopular rules to prevent the abuse of access to our services and to determine the flow and manner in which clients access such services” (SASSA Disability Management Unit, 2020, p. 13). Thus, administrators are instructed that a “booking official” (i.e. the first point of contact for a potential applicant) should probe the client on the availability and location of their medical information. Frontline officials may “request [that] a client … provide documented proof of his/her *medical impairment*” (SASSA Disability Management Unit, 2020, p. 13, emphasis added). In the case of the adult DG, the “client” referred to here would be an adult with a disability seeking access to a grant, while in the case of the CDG, the client would be the primary caregiver of a disabled child.

As noted above, the SGDMM specifically governs the distribution of *disability-related grants*, and any individuals involved in these administrative processes, which fall under the auspices of SASSA’s Disability Management Unit. However, this is not a stable category. In my interviews with regional leadership, it became clear that attempts were previously made to specialise disability-related grant administration but that this plan changed, so all grants are administered by the same frontline workers who are rotated periodically (Trafford and Swartz, 2021). However, SASSA’s frontline staff are not given specific training in recognising or understanding impairment(s) and their impact. This critical gap in training was also highlighted in a 2016 investigation, which reported that, “there is no consistency in the … approach to disabled people and a lack of appreciation for the adjustments that might have to be made … to accommodate the[ir] different needs” (Kidd et al., 2018, p. 74).

Part of the difficulty in providing disability-informed training to SASSA officials seems to be the onerous workload and high tensions associated with the adult DG (G. Kelly, 2013, 2017, 2019). One participant in my study who worked in regional management explained that previously there were “dedicated disability officers” who were responsible for processing disability-related grants at each local SASSA office. This approach was altered some years ago and according to SASSA documentation, staff rotation happens so that SASSA administrators have exposure to and are able to administer any of the country’s numerous grant types. According to my respondent, however, the real reason was the scale and intensity of workload and the “bad reputation” of the adult DG portfolio, which is “a lot of work, so staff kind of shy away from this unit … it’s sometimes a very … *stressful* environment to work in” (SO1, emphasis original). Either way, there is currently no specialisation among administrators involved in processing applications and distributing outcomes.

A high-level DSD official who is disabled and works in social assistance programming eloquently explained the impact of the rotational approach detailed above:

[SASSA] officials don’t even have a basic understanding of what disability’s all about, [so] they tend to exclude … children [who are eligible for the CDG]. Not because the children don’t qualify [but] because in [the officials’] eyes, [these children] don’t fall within this category … most of the time, when you go back [to check eligibility], you find that they actually do qualify … Sometimes the child has got an invisible disability, and just at face value [the mother was] told, ‘No no no, your child doesn’t qualify’ (KI11)

The instructions from the SGDMM that encourage gatekeeping at the frontline (as shared above) might explain why, although doctors are supposed to conduct assessments, caregivers in this study and numerous others have reported being repeatedly turned away by SASSA staff *before* their child had been medically assessed (Dimhairo, 2013; Kidd et al., 2018; Letsie, 2016; Trafford, 2023a). In some cases, then, adherence to the official, “standardised” procedures – which are represented by the guidelines in the SGDMM and are framed as the ultimate solution to fraud – could actually *impede* equitable access to social assistance for disabled children, as these children may not even reach the stage at which a doctor can assess their eligibility.

In summary, widespread government corruption, and an active public discourse thereon, appears to have contributed to the establishment of a relatively rigid disability-related grant administration system, at least on paper, that is designed to tighten or limit access rather than ensuring it is equitable. Indeed, the formal documentation and standardised guidelines that support these grants appear to be more focused on limiting corruption and fraud (an *exclusionary* impetus) than on securing equity (an *inclusionary* impetus), despite ideological commitments to the contrary. This pattern may also be informed by the dominant approach to “improving” service delivery and governance in many contemporary democracies: a hyper-rational, idealised Weberian framework of bureaucracy, which connotes the standardisation and formalisation of procedures with optimal efficiency and fairness (Auerbach et al., 2018; Hodder, 2016).

## 4 Relationships, emotion, and informality in bureaucratic administration

In his book *Emotional Bureaucracy*, Rupert Hodder discusses and reflects on his long-term ethnographic work with civil servants in the Philippines and presents a contrasting perspective to the above-mentioned idealisation of standardisation and formality. In dominant bureaucratic approaches, he explains, informality is commonly framed as “a pernicious feature” (Hodder, 2010, p. 232) in the planning and implementation of civil services and policy. Concerns about the damaging effects of informality within government are especially prominent in countries that have been “characterised [by northern, wealthier countries] as weak or personalistic”, because informality may easily be conflated with self-interested behaviour and framed as the root cause of countries becoming “unstable, fragile, weak, captured, corruptible, inadequate, and unconvincing” (Hodder, 2016, p. 114). Thus, especially in countries battling the after-effects of colonialism but still heavily reliant on international aid and development funding, the solution to the negative impact of informality appears to be to try to “engineer a clean, transparent, and *formal* government” (emphasis added, Hodder, 2016, p. 114). From this perspective, the obvious way to prevent fraud is to formalise administrative procedures and, in so doing, to try to limit the actions of individuals at the frontline so that these adhere to very specific written rules and regulations.

These ideas are apparent in the attempts at standardisation and stricter gatekeeping in SASSA’s SGDMM. Hodder argues, however, that such reforms often end up being “superficial and temporary”, because they are based on “new procedures (or the implementation of existing ones with greater vigor) … elaborate systems of oversight, rigid and exclusive credentialism, and a constant layering of rules, roles, responsibilities and functions” (Hodder, 2016, p. 128) that are instituted from the top down. When these rules and responsibilities are not applied equally within a hierarchy, or when an individual can see that they do not make reasonable sense in the context of their own tacit knowledge. This kind of approach may actually contribute to bureaucratic weakness by encouraging “over-conformity and … gaming [of the system]” (Hodder, 2016, p. 128) among civil servants. Hodder theorises that such “hyper-rigid” procedures may thus unintentionally lead to *more* self-interested behaviour.

One key informant in my study who had worked during ex-President Jacob Zuma’s administration (2009–2018), a period especially associated with the misappropriation of state funds, explained that she had left government because, “there was no real wave of political and administrative commitment [to disabled people] … everything just became about really mediocre, malicious compliance” (KI3). Other respondents also expressed this idea, with reference to the overall situation experienced at the frontline of civil services:

I suppose in the grants space there’s so much potential for abuse, so [officials would] rather stick to the rules – and then if there’s an audit, they can justify the decisions that they’ve made (KI10)

… the way social grants are handled, a lot of persons with disabilities, especially children, fall between the cracks … We’re stuck with, ‘This form must be completed’ and, ‘If you don’t have this small thing, there’s no way that I can help you’ … (KI11)

This “mediocre, malicious compliance” has severe knock-on effects for clients.

The unfortunate reality in South Africa is that the government’s existing structures are too under-resourced and “leaky” to manage the enormous workload associated with social grants spending (Damons, 2024a, 2024b; Gronbach, 2020). In a notable recent example, first-year university students were able to inundate the online application system with hundreds of data queries simultaneously, something that ought not to be possible in a system that is disbursing public funds at such a large scale. These students were also able to identify thousands of false applicants based on simple statistical calculations (Cedras and Gosai, 2024a, 2024b). Without an influx of resources and administrative and technological capabilities, which is unlikely in a time of increasing austerity in public spending, informality will probably continue to characterise many aspects of frontline service delivery in South Africa.

This article adds to a growing body of research that suggests that especially in the global south, informality is not always negative and that while it often limits access, it may in some instances facilitate more equitable access (Auerbach et al., 2018; Berenschot and Van Klinken, 2018). Relationality and emotion are often seen as “enemies” of equity, but at least in some cases, these subjective and personal factors may actually fortify a system. This is particularly the case because South Africa does not have sufficient resources to strengthen assessment and distributive mechanisms in ways that would genuinely increase fairness in the short term. Thus, the potentially constructive power of informality is worth our attention, in pragmatic consideration of whether it could be harnessed to take the country closer to its policy goals.

## 5 The possibilities of “positive” or “constructive” informality, as demonstrated through relationships and empathy

Civil servants in South Africa are well-aware that they are working within an observably dysfunctional system and have little power to make substantial changes. The level of need among the population and the constant spectre of under-resourcing add pressure to their work. In addition, in our interviews, respondents working at the frontline of service delivery suggested that even if they had good ideas about how to improve a system, these would be ignored by the “higher ups”, who tend to live in wealthier areas without daily contact with poverty and the particulars of the administration of bureaucratic processes (Trafford and Swartz, 2021). Respondents working at all levels of government also expressed frustration with the lack of interdepartmental collaboration in managing disability-related service provision and support. Although responsibility for disability is supposed to be shared across all government departments, individuals working in the social development and health sectors felt that they carried this responsibility in full. Overall, government officials’ experiences reflected feelings of fatigue and disempowerment within a profoundly hierarchical system.

Unsurprisingly, civil servants have emotional reactions to working within such a fraught environment. Frontline public servants seem to adopt various approaches to managing the impotence and frustration of working within a “broken” system under considerable public scrutiny. One approach is to avoid any accusation of corruption or wrongdoing by rigidly adhering to “the rules”, a tendency that has reportedly increased over the last decade and has been observed in relation to government procurement systems too (N. Gray, 2021). Reflecting on observations in the Philippines, Rupert Hodder describes this behaviour as the result of “[pressure] for over-conformity … derive[d] from a general defensiveness” that leads to “a need to be able to justify one’s actions and decisions, and to demonstrate accountability” (Hodder, 2010, p. 234).

Another reaction to the relative disempowerment of frontline civil servants is the generalised apathy, or sometimes anger, commonly directed at individuals trying to access overloaded social services. The underlying rationale seems to be: if everyone already thinks I am abusing the system and doing a bad job, I will conform to this expectation and do as little as possible. As Hodder suggests, these situations often result in “existing sets of rules, processes and organisations los [ing] psychological force” (Hodder, 2010, pp. 247–248). When government systems are hierarchical and encourage administrators to police both their co-workers and clients who are seeking their constitutional entitlements, it is likely that civil servants’ desire to go beyond the call of duty will be stunted.

As reflected in public media reportage and in interviews for this study, frontline bureaucratic administrators in South Africa appear to have adopted behaviours and practices to manage their difficult context, some of which reflect the negative impact of informality in under-resourced systems. These negative impacts range from apathy or a lack of interest, to outright discrimination or aggression (Gontsana, 2024; Patrick, 2019; Xulu, 2024). Others have also investigated the problematic effects of gatekeeping by SASSA officials involved in the daily administration of all grants in South Africa (M. Du Toit and Lues, 2022), and by health workers who mediate access to public sector health services for the population (Gaede, 2016; Nothdurfter and Hermans, 2018; Walker and Gilson, 2004). In the minimal literature focused specifically on the CDG, including my own prior publications from this study, family caregivers detailed their long and difficult journeys in trying to gain access (Dimhairo, 2013; Letsie, 2016; Martin et al., 2014; Trafford, 2023a). It is certainly critical that such destructive patterns are documented, to draw attention to unconstitutional failures in service delivery.

In this article, however, I offer a complementary idea: that at least *some* informal practices, particularly those that are built on the development of independent and autonomous relationships, have the potential to facilitate improved access, better client service, increased efficiency, and/or the accomplishment of policy goals. For frontline SASSA grant administrators, grant applications and related procedures are the bulk of their responsibility, in a job that is subject to considerable public and media criticism. As such, it may feel like their only power is to mediate access to grants, manifesting in the harsh style of gatekeeping described by caregivers trying to gain access to the CDG. However, in this study and in my prior experiences with under-resourced frontline public sector health workers, alternate practices also emerge. SASSA officials and other public servants interviewed in this study, for example, told stories that demonstrated a strong commitment to their clients, and to developing important partnerships with other government entities to improve service delivery. Stories of uncaring and uncommitted public servants are real, but they are also not representative of everyone working within these structures.

## 6 What does constructive informality look like in practice?

Informality is important because it is often “the material from which new practices emerge and around which [a] formal organisation is shaped, re-shaped and improved” (Hodder, 2010, p. 245). As one of Hodder’s respondents explained while reflecting on their professional environment as civil or public servants, it is preferable when “[structures] evolve organically … because then … people … feel that they are part of the decision-making itself, and it is not something imposed” (Hodder, 2010, p. 246). This kind of behaviour represents something that Hodder calls “positive informality”, a term I have slightly adapted and called “constructive informality”, to imply its potential to contribute to (rather than only detracting from) social justice. I define constructive informality as the creative ability of individuals working within resource-scarce bureaucratic systems to observe and make the best use of their relationships to achieve better outcomes, or to make decisions that fall outside of their formal role or guidelines, but which improve clients’ interactions with the state. In this way, public sector workers and civil servants are applying a kind of “flexibility, negotiation, or situational spontaneity” (Boudreau and Davis, 2017, p. 155). By taking examples of this kind of bottom-up practice seriously, and thinking about what might work to harness “constructive” informality in the system as it *actually* operates (rather than as it *ought to* operate), we may gain new insights about how to motivate fatigued and fearful public servants. In so doing, the core *intentions* of progressive policy (the inclusion and upliftment of disabled people) may be better served, instead of getting trapped in perfecting the *methods* for their application.

Below, I provide a series of vignettes from respondents within or directly associated with SASSA, to illustrate how this kind of constructive informality can operate. These narratives demonstrate its hyperspecific configuration, which is often facilitated by direct interpersonal relationships, a significant but often neglected element of bureaucratic systems strengthening. Further research that interrogates and tests these ideas in different circumstances is much-needed and very welcome.

### 6.1 Fadilah

Fadilah works as a clerk at a SASSA regional office. Her day-to-day responsibilities include data capture and the verification of payment claims. As noted above, these roles are not specific to a particular grant type but one of her key roles was to liaise with SASSA’s directly-contracted doctors who conduct eligibility assessments for the adult DG and child CDG. In 2021, she had worked at SASSA for about five years, before which she had been a recipient of the DG after acquiring a disability in adulthood. Fadilah grew up and still lived in a deprived area, and many of her neighbours also received grants. As a recipient of the DG herself, Fadilah had encountered various government officials who introduced her to disability-related training programmes, or opportunities for job progression. This series of chance (informal) connections, as well as her own personality and drive, had helped her to obtain care for her disability and a more secure economic position. However, these interactions were not embedded into the formal referral and support system and therefore, were not consistently available to others seeking disability-related support.

In our interview, Fadilah specifically mentioned the importance of strong relationships and how her prior work experiences had enhanced her approach to her job. Previously, as a volunteer with the South African Police Service (SAPS), Fadilah had learned how to follow SAPS’ internal chain of command and identify the right teams to despatch in response to alerts. When sending doctors and SASSA officials to areas with a particularly high-risk of violence, she used her personal network to protect them:

[W]hen we know the areas that we [are going to] do assessments, I will call [SAPS] … because I have a relationship with [them] … I ask them to inform [the nearby team] … And if it’s a [crime] hotspot, [SAPS] will go in with [our team] … we need to look after our officials and our doctors

Hodder explores similar ideas in his description of how the inter-organisational or interdepartmental movement of civil servants allows them to gather “an extensive, complex and informal network of relationships through which inter-agency business is conducted … [and which] provide an important sense of cohesion and means of coordination” (Hodder, 2010, p. 240). Although Fadilah is working at a lower hierarchical level than the civil servants Hodder interviewed, her insights are similar. Her specific tacit knowledge of the areas in question are brought to bear on her current duties in informal, but constructive, ways.

Fadilah brings a deep understanding of the *context* in which she is working to her bureaucratic job. By safeguarding the doctors who do eligibility assessments, she is protecting an extremely scarce resource for SASSA. The SGDMM explicitly states that the “[p]hysical safety of disability assessors at assessment venues” (SASSA Disability Management Unit, 2020, p. 1) is a key challenge in the management of disability grants, and that a high turnover of skilled assessing doctors causes assessment backlogs for the population. Thus, while Fadilah’s formal job description includes duties such as invoicing, scheduling, and data capture, her *actual* job involves a range of “informal” tasks too, many of which are in the realm of relational work. Encouraging or forcing her to leave her personal emotions and relationships out of her work would likely detract from, rather than strengthen, the quality of this work.

### 6.2 Dr Stewart

Dr Stewart is a specialist paediatric doctor who works at a public hospital in one of South Africa’s largest cities. Dr Stewart has extensive experience in assessing eligibility for the CDG and working with disabled children and their families. Also reflecting on the importance of relationships and informal practices, she explained that she and her colleagues had independently developed an efficient multidisciplinary team, with strong links to the provincial Department of Education. Having previously struggled with intersectoral collaboration, these teams worked together once on a shared project, and this helped them to establish a collegial relationship. The relationship was then sustained with regular meetings:

… if [the Education team is] anxious about a particular child, they will often ask us to sort it out or intervene, and we can do that. There is that loop, which is unusual, because I don’t think a lot of the other medical groups … have that sort of relationship with Education.

However, Hodder reminds us that while informal connections and networks can be “stable and reliable”, they may also “prove very fragile” (Hodder, 2010, p. 240) because of a reliance on a particular individual for their sustenance. Dr Stewart expressed a similar sentiment, explaining that despite their progress, if one or more of the people involved in this collaborative relationship left their position the team might falter or dissolve. Especially in resource-constrained environments, one person’s behaviour can be massively impactful. This means that a particularly well-run or well-managed public service offering may rely more heavily on a specific individual or small group than it would in other settings.

Clearly, there is a critical element of the work needed at the interface between the public and state organs that cannot be included in a manual or within a formal job description – but it *should* be identified and studied closely, to draw out important characteristics that may help to strengthen systems for state support in ways that are pragmatic and responsive to reality.

### 6.3 Veronica

For Veronica, who worked in inclusive education at the provincial Department of Education, personal relationships had facilitated a strong foundation for her work, even though these were not formally mandated or facilitated. These relationships had helped intersectoral teams to bypass many difficulties because of the trust they had previously established:

[An official in a different department] was a school doctor when I worked in the district, so we got to know each other through … another project … the more people work together, the more you get that blurring of boundaries, which leads to more coordinated working … Before this happened, it was about budgets: ‘Whose budget is going to fund this [project]?’ … something [else that] also gets in the way of service delivery is that you don’t understand how the bureaucracy works in another department … [but]once you get an entrée into the department, you start to figure out, ‘Oh ok, this is the person that can help me with this issue’ … we all know each other now, it’s become quite collegial.

This approach had helped Veronica and her collaborators to progress past their initially oppositional attitudes, making them more likely to achieve their policy-related goals.

Following a period of ethnographic work in the South African Department of Home Affairs, Colin Hoag explained that when service delivery bureaucracies become overly complex or opaque, they are rendered “illegible” to the very populace they were appointed to serve (Hoag, 2010). The narratives of government officials in my study suggest that unless strong relationships are proactively built, preferably around shared goals, government departments that are already impenetrable to clients seeking their support may also become “illegible” to one another. This illegibility can breed mistrust or conflict, making it harder to collaborate across departments, regardless of high-level guidelines that may recommend intersectoral collaboration. Veronica’s narrative describes how this problem can be addressed by independently building personal relationships and taking advantage of the trust developed therein.

### 6.4 Michelle and Tabitha

Michelle, a non-disabled social worker based at a large urban public sector hospital is regularly involved in connecting patients at the hospital with doctors who can conduct disability-related grant assessments. She felt that it was critical that all public servants introduce themselves to clients, as a signifier of personal responsibility:

… [SASSA] officials don’t give their names … And that’s a big problem. Because I think it’s a basic … way of showing respect, identity, and professionalism … when you’re working with a client, you should introduce yourself … There should be no fear in giving your name if you’re going to do the process appropriately … it’s almost an anonymous encounter that the family has at the SASSA office, they’ve got no one that they can turn back to and say, ‘So-and-so dealt with my process the first time I went there. The second time I went there, Whatshisname helped me.’ It empowers the family … And it’s a personal process then, you connect with the person.

For Michelle, introducing oneself to a client showed that you were taking personal responsibility for your work, partly for accountability reasons but also to humanise the person seeking services. Michelle also described how she had experienced these impersonal interactions with SASSA officials herself too, suggesting that this combination of anonymity and apathy represented a resistance to building professional intersectoral relationships. The reluctance of SASSA officials to give their names may be a “lazy” or apathetic response, but it may also be another dimension of their affective reactions to the environment within which they work. In other words, the fear of being accused of impropriety might inform decisions to stay anonymous.

Finally, Tabitha, who manages a provincially-funded community-based support facility that primarily serves children and young people with complex disabilities and is often involved in facilitating access to disability-related grant applications and eligibility assessments, argued that service delivery for disabled people worked best when there was strong disability representation in government:

… it really helped when DSD started employing people with [disabilities] themselves … [who] were put into managing teams there, and they could say, ‘Well, this is a need that I have, and this is what I feel would help me, so I feel it will help others’

Tabitha believed that the *personal* experiences and feelings of these disabled government employees helped them to implement more accessible and inclusive processes.

## 7 Learning from constructive informality

Accepting informality in systems for public service delivery can be frightening, because this feature is closely connoted with real problems of corruption and misuse of power. However, as Hodder argues, “enmeshed around” these pernicious phenomena, other features may also occur, such as “professionalism, technical competence, imagination, creativity, realistic compromise, commitment, and a willingness to take risks and suffer the consequences” (2017a, p. 15). Indeed, these constructive aspects of informality may be the same characteristics that “keep the civil service and the organs of government working” (Hodder, 2017, p. 15), especially in resource-limited environments. When the human elements of bureaucratic work are stripped away, however, the capacity for flexibility, creativity and empathy among the people who do this work may also be constrained.

While not necessarily generalisable, the vignettes shared above suggest that instances of informality are not *exclusively* destructive and may, at least in some cases, assist in improving service delivery. In another example from the literature, authors of a 2016 study noted that Down syndrome was not well-understood by medical assessors or SASSA staff but that in one SASSA local office, a particularly committed official had a child with Down syndrome and had improved her colleagues’ understanding of the condition. This local office “[now had] a reputation for treating children with Down syndrome sympathetically and to a high standard” (Kidd et al., 2018, pp. 73). In my study too, participants who were especially motivated and committed to attaining equity for disabled people usually had a pre-existing personal or professional interest in disability. Some were trained social workers or psychologists who had moved into government administrative positions, while others had had a personal relationship with a disabled child or adult that had encouraged them to become involved in academia or advocacy around disabled childhoods.

However, the environment within which frontline bureaucrats are operating does not seem to encourage their understanding of or empathy for the specific needs of disabled people and their families. Some of these officials acknowledged the widespread lack of disability-awareness, especially in client-facing positions at SASSA, and expressed a desire to “do more” about this. One official explained to me that although she was permitted to hold informational workshops where disabled people could, for example, share their experiences with SASSA administrators to improve understanding, no budgets were dedicated to this work. This suggests that some may want to do more but feel hamstrung by their environment and its constraints, and reminds us that it is important to distinguish between “a public official who has simply never thought of adapting form-filling procedures to accommodate [disabled people’s] needs … and another official who believes that people with [disabilities] … should not be out in society” (Swartz, 2012, p. 37).

Similarly, a respondent in this study who is an academic and wheelchair-user reflected on research on disability-friendliness in South Africa’s civic court administration, which showed that “at ground level, staff were generally willing, kind, and happy to be involved – but they need guidance, and they need support” (KI2). It is possible that a lack of empathy may reflect ignorance or lack of exposure, rather than intentional discrimination. Training programmes to harness informality and encourage constructive behaviour (rather than “mediocre, malicious compliance” with formal guidelines) could be framed as professional development, an approach which has improved commitment to and investment in working with people with disabilities in other contexts (Igei, 2020; J. Kelly et al., 2022). While some may always be resistant to expanding their worldview, or may consider training programmes “extra work”, others could benefit from being reminded of the critical social *value* of their work. This might help them to find additional energy and gain an interest in performing these important jobs well, and with care.

## 8 Conclusion

In the Philippines (Hodder, 2010, 2017a), Brazil and India (Boudreau and Davis, 2017), Indonesia (Berenschot and Van Klinken, 2018), the UK (Hodder, 2016) and even Sweden (Graham, 2002), emotions and social relationships (the foundations of informality) clearly play a significant role in the bureaucratic implementation of policy and the interactions between citizens and their government. In South Africa, this emotionality is strongly flavoured by justified public mistrust in government, interdepartmental and intersectoral tensions due to competition over limited government resources, and a top-down approach to decision-making. Part of the difficulty with reforming the delivery of social assistance is that, in the minds of those who design these procedures, being *human* seems to be conflated with being corrupt. From this perspective, an inevitable part of what it means to be a person is to be susceptible to corruption and so, the only way to eradicate corruption from the distribution of state-funded benefits is to remove human judgements from these procedures. This approach is also reflected in international discourses around welfare benefits, especially over the last two to three decades, which have rejected informality and subjectivity in favour of formality and (attempts at) impartiality. However, this kind of “procedural” objectivity seems to provide only “superficially plausible, but ultimately specious” solutions to “longstanding anti-welfare tropes” (Porter et al., 2021, p. 279).

While discussions about how corruption can thwart service delivery are well-documented, fewer researchers have considered how fears about impropriety in service delivery may unintentionally detract from the implementation of progressive policies. A preoccupation with limiting government fraud appears to have constrained equitable access to, and the design of systems for, social assistance for disabled children and their families in South Africa. This is despite these grants being a constitutional entitlement and, for many families, the only reliable source of state support and one that shapes their access to all other critical social services. My own prior work and the work of others has established that guidelines for access to and the administration of the CDG must be clarified so that government can be held to account for non-delivery. As such, the intention of this article is not to argue that we should *increase* informality in under-resourced bureaucratic systems, nor that we should remove any well-functioning checks and balances that genuinely prevent illegitimate access to important state benefits.

However, as evidenced in this article, some civil servants exercise *constructive* informality in their work, using their own personal networks and lived experiences to go beyond the call of duty. For these respondents, their subjectivity and capacity to build and sustain relationships were an asset to their professional duties, even though these decisions and behaviours may not have been supported by official guidelines or included in formal job descriptions. In treading this path, I echo Hodder’s warning that the delegitimization of informality may result in the suppression of “creativity and experimentation”, because “if one is to behave outside the constraints of formality, then why risk having one’s behaviour misinterpreted for the sake of a more effective organisation rather than personal gain?” (Hodder, 2010, p. 245). This reaction to working within dysfunctional, hyper-scrutinised environments is represented at the frontlines of South African public service delivery, where public servants appear to shirk responsibility by performing the narrowest, most limited version of their jobs.

My findings in relation to the CDG support and extend Kelly’s contention that in trying to tighten up the definition of disability and aim for more objective assessments, “the political and public debate has lost sight of the objectives of the DG”, which has “distracted policy makers from developing a DG system that promotes the inclusion and development of disabled people in society” (Kelly, 2016a, pp. 69-71). These goals are included in high-level policies and commitments but there appears to be no investment in deepening frontline government workers’ understanding of the manifestations and implications of disability. Instead, their work is reduced to a series of administrative steps, with ever increasing hierarchies and scrutiny, to try to reduce corruption.

Obviously, a one size fits all approach to relationship-building is not feasible; functional arrangements are always specific to the people and circumstances involved. However, paying attention to narratives that demonstrate why and how constructive informality works well may help us understand how best to develop and sustain these important networks and relationships, ultimately improving service delivery for disabled people. Most importantly, the disability-related grants portfolio should be reframed so that public servants feel that they have a critical role to play in delivering life-saving income support. This may increase satisfaction from professional duties, with potentially positive effects on applicants’ experiences with SASSA.

## Data Availability

The data that has been used is confidential.

## Data statement

The data that informed this article were collected under strict conditions of anonymity and confidentiality so transcripts cannot be shared in full. However, de-identified sections of the data can be provided on reasonable request to the author.
